# *Notes from the Field:* House-to-House Campaign Administration of Inactivated Poliovirus Vaccine — Sokoto State, Nigeria, November 2022

**DOI:** 10.15585/mmwr.mm7247a3

**Published:** 2023-11-24

**Authors:** Oladayo Biya, Jibrin Idris Manu, Joseph C. Forbi, Gatei wa Nganda, Hadley Ikwe, Adamu Sule, Aboyowa Edukugho, Abba Shehu, Nurudeen Aliyu, Nyampa David Barau, Eric Wiesen, Roland W. Sutter

**Affiliations:** ^1^Global Immunization Division, Center for Global Health, CDC; ^2^African Field Epidemiology Network, Abuja, Nigeria.

After the 2015 documentation of global eradication of wild poliovirus type 2,* Sabin type 2 oral poliovirus vaccine (OPV) was withdrawn from routine immunization (RI) in all OPV-using countries in 2016, in a global synchronized switch from trivalent OPV (containing vaccine virus serotypes 1, 2, and 3) to bivalent OPV (containing serotypes 1 and 3), to reduce the rare risks for type 2 vaccine-associated paralytic poliomyelitis. Concurrently, the Global Polio Eradication Initiative (GPEI) recommended that all OPV-using countries introduce ≥1 dose of inactivated poliovirus vaccine (IPV) into RI programs; IPV protects against paralysis caused by all three serotypes but cannot be transmitted from person to person or cause paralysis. Use of OPV, especially in areas with low vaccination coverage, is associated with low risk of emergence of vaccine-derived polioviruses (VDPVs). As susceptible persons in new birth cohorts accumulated after withdrawal of OPV type 2, population immunity against infection with serotype 2 declined (*1*), facilitating the emergence of circulating VDPV type 2 (cVDPV2). During the previous 7 years, cVDPV2 outbreaks required response supplementary immunization activities (SIAs) with monovalent type 2 OPV (mOPV2); however, if SIAs were not of sufficiently high quality and did not achieve high enough coverage, new emergences of cVDPV2 occurred.

## Background

Routine administration of 1 dose of IPV at age 14 weeks, which was recommended by GPEI following the switch, provides protection against paralysis caused by all three poliovirus serotypes to approximately 60% of recipients (*2*); however, 1-dose RI IPV coverage is low in many countries. A substantial number of subnational jurisdictions in Nigeria reported RI IPV coverage <50%, including many in the northern part of the country, based on a combined National Immunization Coverage Survey and Multiple Indicator Cluster Survey conducted in 2021^†^ to assess vaccination coverage and various aspects of children’s health and education.

Controlling cVDPV2 outbreaks requires conducting multiple SIAs. In 2021, novel OPV2 (nOPV2), a more genetically stable version of OPV2 that is less likely to revert to neurovirulence in settings of low population immunity, replaced mOPV2 (*3*). However, if these campaigns do not reach a high proportion of resident children, cVDPV2 circulation could continue. In Nigeria’s northwest Sokoto State, outbreak transmission continued even after eight nOPV2 SIAs conducted since March 2021 (National Primary Health Care Development Agency, Polio Expert Review Committee meeting, Abuja, Nigeria, unpublished data, 2023). Because Sokoto reported 27% RI IPV coverage in 2021 (Figure), a campaign to increase IPV coverage was planned. To conserve limited IPV resources, a 2-dose fractional-dose IPV (fIPV) series, which consists of an intradermal injection of one fifth of a full intramuscular IPV dose, can be administered instead of a singular intramuscular dose (*4*). The 2 doses are administered at an interval of ≥4 weeks. A large SIA with fIPV administered at fixed-post immunization sites has been implemented in Pakistan, with coverage of 85% (*5*).

**FIGURE:**
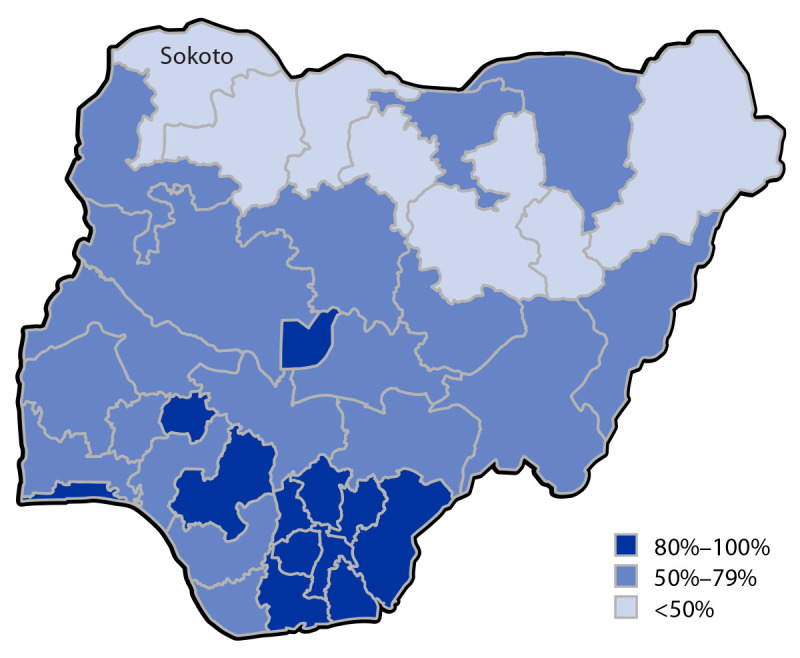
Inactivated poliovirus vaccine 1-dose coverage, by state — National Immunization Coverage Survey and Multiple Indicator Cluster Survey, Nigeria, 2021

## fIPV Vaccination Campaign and Postcampaign Coverage Survey

To evaluate whether fIPV could be administered in a house-to-house campaign using a needle-free jet injection device (Tropis, Pharmajet^§^), a pilot project was conducted in Sarkin Adar Gidan Igwai, a ward (subdistrict) of Sokoto State. One fIPV dose was added to an already planned nOPV2 SIA in November 2022, targeting children aged 3–59 months. Nurses were trained to use the devices before they were deployed with nOPV2 vaccination teams. The fIPV dose was withdrawn from a multidose vial into a cartridge in each home. Field evaluation conducted at the time of fIPV vaccination documented that a majority of parents (94%) and health staff members (93%) preferred needle-free injections over the customary needle and syringe administration. This activity was reviewed by CDC, deemed not research, and was conducted consistent with applicable federal law and CDC policy.^¶^

To assess postcampaign fIPV coverage, a survey was conducted using the World Health Organization modified cluster survey technique to sample 210 children aged 3–59 months from 30 settlements in the pilot ward. The coverage survey indicated that 87% of children in the target age group had received fIPV during the campaign.

## Preliminary Conclusions and Actions

This pilot study demonstrated that administering an injectable vaccine in a house-to-house campaign with needle-free jet injector devices is feasible and can achieve high coverage. Intensification of RI, including increasing immunization sessions, provision of supportive supervision, and ensuring vaccine availability, will be needed to complete vaccination of children in the pilot ward with the second fIPV dose. Additional pilot studies targeting larger populations should be conducted before this approach can be applied in other low-IPV coverage areas.

## Footnotes

*
https://polioeradication.org/news-post/global-eradication-of-wild-poliovirus-type-2-declared/

†
https://www.unicef.org/nigeria/media/6316/file/2021%20MICS%20full%20report%20.pdf

§
https://pharmajet.com/tropis-id/

¶ 45 C.F.R. part 46, 21 C.F.R. part 56; 42 U.S.C. Sect. 241(d); 5 U.S.C. Sect. 552a; 44 U.S.C. Sect. 3501 et seq.
